# Case of Male Twins United from the Axilla to the Spine of the Ileum

**Published:** 1824-04

**Authors:** William Toms

**Affiliations:** Member of the Royal College of Surgeons; Licentiate of the Society of Apothecaries, &c.


ART. VI.—*Case of Male Twins united from the Axilla to the Spine of the Ileum.* Communicated by WILLIAM TOMS, Member of the Royal College of Surgeons; Licentiate of the Society of Apothecaries, &c.

A CASE of midwifery occurred in this neighbourhood a short time since, of rather an uncommon description. A woman was delivered of full-size male twins, perfectly united from the axilla to the spine of the ileum, and facing each other; they weighed thirteen and a quarter pounds; measured from head to foot twenty inches, and ten inches across the hips; externally they were complete in every respect, (excepting there being but one umbilicus,) having four arms, four feet, &c. &c. On dissection, it was found there was but one heart, one spleen, one jejunum, and one pancreas. I believe there is no case on record with these peculiarities; therefore have been induced to forward you the following particulars of this interesting case.

Joanna Masters, a thin and delicate woman, thirty-six years of age, residing at Batson, near this town, was taken in labour on Monday, the 9th of February last, at nine o'clock in the evening. A midwife was, as usual, sent for, who arrived at half-past ten: at this time the motion of the children were distinctly felt. Pains still increasing, and the os uteri sufficiently dilated, the head was soon delivered; although the most violent labour-pains continued, she could not bring down the shoulders. In this state the patient was kept, with the neck of the child completely wedged in the vagina, until a quarter of an hour after twelve, expecting each succeeding pain would bring away the child. The husband, being at this time much alarmed for the safety of his wife, instantly came for my friend and preceptor, Mr. William Ilbert Pearse, who arrived there at half-past one; the distance from hence being about four miles. He found the head, as before described, completely delivered, with the neck tightly embraced in the vagina; the face and head were much enlarged, discoloured, and distended with blood: he immediately gave her thirty drops of tincture of opium, and, by dilating the soft parts with his fingers, gradually brought down the shoulders and arms. All difficulty was then supposed to be over; but, much to his astonishment, he found, on further examination, a second child in utero, firmly adhering to the thorax and abdomen of the other, with the left leg presenting, which was immediately returned. The size of the children being considerably larger than was possible to pass through the vagina, without laceration of the perineum, or diminishing the bulk of the children, my attendance was requested, and a messenger dispatched for me, by the request of Mr. Pearce; desiring I would also send a perforator, catheter, &c. &c. I hastened to the spot; and, on my arrival, learnt the most alarming symptoms had taken place about half an hour previous, and the patient sinking from debility. Mr. Pearse found it indispensably necessary to perforate the chest and abdomen of the second child with his fingers, which considerably diminished the bulk: the legs of the first child were then delivered, the head of the second brought down, and delivery accomplished without the aid of any instrument. By desire of Mr. Pearse, I delivered her of the placenta, which firmly adhered to the fundus uteri: this was easily removed, by introducing my left hand into the uterus, and grasping the abdomen with my right. Very little hemorrhage ensued, and the uterus gradually contracted. I found, on examination, there was not the least laceration of the perineum, or any of the soft parts. The placenta (there being but one,) was of the common size ; the funis much smaller than usual. The following mixture was ordered:

℞. Sperm. Ceti, ʒ ij.

Ovi Vitellus unius

Spirit. Æther. Nitr. ʒ iij.

Tinct. Opii, gr. xl.

Aquæ Cinnam. ℥iv. M. Fiat mistura capiat cochleare magnum 4tis horis.

Vespere.—Found she had been complaining of slight pains in the abdomen and uterus ; discharge from the latter regular ; pulse 90. Persistat in usu misturæ.

From this time she gradually recovered, and, by the 23d, was able to resume her usual avocations.

The woman has had four children before: the first and third labours were lingering and difficult, but did not require the assistance of a surgeon.

The following circumstance occurred during the fourth month of her pregnancy:—A man, who gains his living by exhibiting two wax figures of children united together, (precisely in the same way to those she was delivered of,) came to her house, and wished her to see them ; but, being in the family-way, and having previously heard a full description of them from her neighbours, she considered them too frightful for her to view, and refused. She also received a severe blow in the abdomen about the same time from a fall, and never felt well from that period to her delivery. I am well aware these circumstances could not participate in uniting the children in utero, at her advanced stage of pregnancy ; but merely mention it as a curious coincidence.

On Wednesday, the 11th of February, (the morning after delivery,) I commenced the dissection, assisted by Messrs. Stone and Mills, in presence of Dr. Bridgman and several other gentlemen.

*External appearances.*—The child first delivered was larger than the other, particularly the head, which was considerably swollen ; the face and neck put on a livid appearance, as well as the arms and feet ; blood being extravasated under the skin in those parts.

*Brain.*—On opening the head of the child first delivered, the brain was found extremely turgid with blood ; and, on separating the hemispheres, a large quantity escaped ; the lateral ventricles much enlarged, and distended with coagulated blood.

Nothing peculiar was observed in the other, although the brain was firmer than usual.

*Chest.*—The lungs throughout were of a bright grey colour, and floated on being removed and placed into water; the pulmonary vessels extremely turgid with blood. There was but one heart, which was situated immediately between both children, at their junction: it was of a peculiar oblong shape, which at first sight put on the appearance of two united in one pericardium. On examination, we found it but one, with only one set of vessels leading immediately to and from it; the distribution of which could not be traced so minutely as was wished, from the laceration which took place on delivery, and other circumstances; Mr. Pearse being unwilling we should destroy the union, it being his intention to preserve the children united. The foramen ovale was open. The thymus glands varied materially in size, although the colour (a pale red) was the same; one weighed 116 grains, the other only six. The pleuræ much inflamed, but did not adhere to the lungs.

*Abdomen.*—The livers were very large and healthy. On tracing the umbilical vein into the venæ portæ, we found it distributing branches to both livers, which were united at their base. The ductus venosus terminated naturally in the venæ cavæ abdominalis, but rather contracted and pervious. The gall-bladders were large, and contained healthy bile. The spleen (there being but one, and that in the child first delivered,) was large, and of a dark hue. Pancreas natural. The kidneys in both were highly inflamed throughout, and distended with coagulated blood; the bladders also partook of the same blush.

*Stomachs.*—Nothing peculiar was observed in either; but, on tracing the duodeni, we found them contracted and small,* but terminating in one about the extent of three inches from the pyloric orifices, and continued but one canal until it arrived at the termination of the jejunum, (where there was a firm adhesion to the umbilicus,) which then bifurcated into the ilii, and continued to their natural terminations. The intestines were much inflamed, and contained a large quantity of meconium.

From the colour and buoyancy of the lungs, turgid state of the pulmonary vessels, and contracted appearance of the ductus venosus, we have some reason to suppose the children must have breathed. They are preserved in their united state, and remain in possession of Mr. Pearse.

***Kingsbridge, Devon; 7th March,* 1824.**

---

*** The right of which formed a strong adhesion to the under surface of the liver (of the child first delivered), near the neck of the gall-bladder.**

