# Errata in Last Number

**Published:** 1824-05

**Authors:** 


Errata *in last Number.*

Page 293, line 45, begin a new sentence with the words "By desire, &c."—and p. 294, line 36, for *Mills* read *Wills*.

